# The Effect of Microwave and Radiofrequency Ablation (MWA/RFA) on Liver Volume in Patients with Primary and Secondary Liver Tumours: A Retrospective Analysis

**DOI:** 10.1007/s00270-023-03503-0

**Published:** 2023-07-10

**Authors:** Robrecht R. M. M. Knapen, Remon Korenblik, Sinead James, Glenn Dams, Bram Olij, Sanne W. de Boer, Ronald M. van Dam, Christiaan van der Leij

**Affiliations:** 1 Department of Radiology and Nuclear Medicine, Maastricht University Medical Center+, Maastricht, The Netherlands; 2 CARIM, School for Cardiovascular Diseases, Maastricht University, Maastricht, The Netherlands; 3 Department of Surgery, Maastricht University Medical Center+, Maastricht, The Netherlands; 4 GROW, School for Oncology and Reproduction, Maastricht, The Netherlands; 5 Department of Radiology and Nuclear Medicine, Zuyderland, Sittard, Heerlen, The Netherlands; 6 Department of General, Visceral and Transplant Surgery, University Hospital Aachen, Aachen, Germany

**Keywords:** Microwave ablation (MWA), Radiofrequency ablation (RFA), Liver volume, Ablation, Future liver remnant (FLR), Hypertrophy

## Abstract

**Purpose:**

It is known that thermal liver ablation can induce liver hypertrophy. However, exact impact in liver volume remains unclear. The aim of this study is to assess the influence of radiofrequency or microwave ablation (RFA/MWA) on liver volume in patients with primary and secondary liver lesions. Findings can be relevant in assessing the potential extra benefit of thermal liver ablation in preoperatively performed liver hypertrophy inducing procedures, such as portal vein embolization (PVE).

**Methods:**

Between January 2014–May 2022, 69 invasive treatment naïve patients with primary (*n* = 43) or secondary/metastatic (*n* = 26) liver lesions (in all segments, except in segments II/III) treated percutaneously by RFA/MWA were included. Total liver volume (TLV), segment II + III volume (serving as “distant liver volume”), ablation zone volume and absolute liver volume (ALV, calculated by subtracting the ablation zone volume from the TLV) were the study outcomes.

**Results:**

ALV in patients with secondary liver lesions increased to a median percentage of 106.87% (IQR = 99.66–113.03%, *p* = 0.016), volume of segments II/III increased to a median percentage of 105.81% (IQR = 100.06–115.65%, *p* = 0.003). ALV and segments II/III in patients with primary liver tumours remained stable, with a median percentage of 98.72% (IQR = 92.99–108.35%, *p* = 0.856) and 100.43% (IQR = 92.85–109.41%, *p* = 0.699), respectively.

**Conclusion:**

In patients with secondary liver tumours, ALV and segments II/III increased after MWA/RFA by an average of approximately 6%, while ALV in patients with primary liver lesions remained unchanged. Besides the curative intent, these findings indicate the potential added benefit of thermal liver ablation on FLR hypertrophy inducing procedures in patients with secondary liver lesions.

**Level of evidence:**

Level 3, non-controlled retrospective cohort study.

**Graphical abstract:**

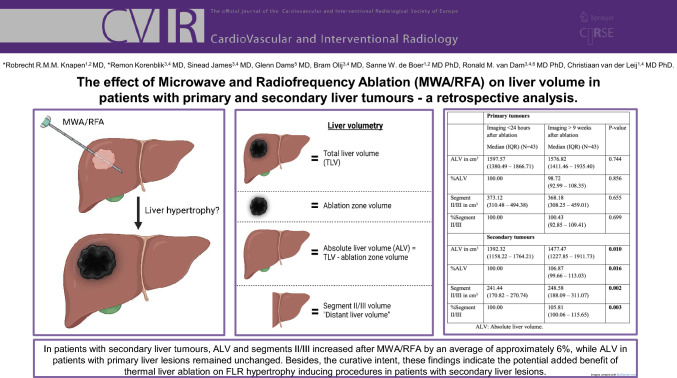

## Introduction

Thermal ablation is an effective first-line treatment for patients with a primary or secondary liver carcinoma [1–3]. It is a versatile technique with a low complication rate and a short hospital admission compared to surgery [4, 5]. A recent mice study has demonstrated that the loss of hepatocytes due to radiofrequency ablation may lead to a proliferation in total liver volume and regeneration of the liver parenchyma, with a maximum of 9.4% at 10 weeks after ablation [6]. The effect of ablation on the liver volume of human livers is, however, still not well known.

In patients undergoing major liver surgery (for example in diffuse liver metastases), fatal post-hepatectomy liver failure (PHLF) continues to be a problem [7, 8]. Previous research has shown that up to 30% of patients undergoing major liver resections (resection of at least three segments) develop different grades of PHLF, making it the largest cause of mortality after liver surgery [9]. PHLF is primarily caused by a low functional capacity (due to cirrhosis, cholestasis) or an insufficient future liver remnant (FLR) [4]. In order to stimulate growth of the FLR, often interventions such as a portal vein embolization (PVE) or associated liver partition and portal vein ligation for staged hepatectomy (ALPPS) are performed 2–6 weeks before liver resection [5, 10–12]. However, following these interventions FLR hypertrophy still remains insufficient in 20–30% of cases to allow safe surgery. The need for other or additional hypertrophy inducing interventions therefore remains. The findings in animal research underline the potentially added value of liver ablation in inducing liver hypertrophy.

The aim of this study is therefore to retrospectively assess liver volume changes after ablation in invasive treatment naïve patients, to evaluate the potential additional effect of this technique on liver hypertrophy inducing procedures.

## Materials and Methods

This is a single-centre retrospective cohort study for patients receiving percutaneous ablation of primary or secondary liver lesions in a tertiary care hospital in the Netherlands. The study protocol was reviewed by the local medical ethics committee. The need to obtain individual informed consent according to the Dutch Medical Research Involving Human Subjects Act was waived. This study was conducted in accordance with good clinical practice and the applicable national and European laws.

All required data were collected from the hospital’s electronic health records (SAP GUI 7.60) and recorded in an online electronic database (Castor EDC, V2020.2.20). All patient information was pseudonymized by using a unique code, which was saved on a local external hardware disc of the hospital that was only accessible to the associated researchers.

### Patient Population

All consecutive patients with primary or metastatic liver lesions, treated by percutaneous MWA or RFA, between January 2014 and May 2022, were included. Other inclusion criteria were: A CT scan or MRI scan less than 24 h after ablation, and a CT or MRI scan at least 9 weeks after ablation. Exclusion criteria were: Patients with previous ablations or liver surgery, ablations performed during a (laparoscopic) liver resection and ablations in liver segments II/III.

For ablation, the HS Amica generator (HS; Hospital Service) or the NeuWave Microwave ablation system (Ethicon; Johnson & Johnson) was used, with needle placement under ultrasound and/or CT guidance. The duration of the ablation and the used wattage depended on the required ablation zone volume and underlying disease and was determined by the treating physician.

### Liver Analysis

All CT scans were analysed using the Syngo.via workstation (Siemens Healthineers GmbHs, Client 5.1; liver analysis programme). MRI liver volumetry assessment was performed using the OsiriX (DICOM Viewer Lite, v.12.5.3). Liver volumetry, using manual segmentation, was performed by a trained researcher and supervised by an experienced interventional radiologist. To train the researcher, twenty liver volumes and segmentations were performed together with the researcher and the interventional radiologist, before liver volumetry of the study population was performed.

### Outcome Measures

Total liver volume in cm^3^ (TLV) and volume of liver segments II/III were measured directly or within 24 h after the ablation and after > 9 weeks. The timepoint of > 9 weeks was chosen, since maximal growth is expected in the first 9 weeks [6]. The volumes of segments II/III were regarded as distant liver segments and surrogate for FLR, to compare local and distal volume changes. To reduce measurement errors, only segments II and III were selected as their anatomy allows reliable delineation.

The ablation zone volume was measured on the CT or MRI scan < 24 h after ablation. Absolute liver volume (ALV) was calculated as TLV minus ablation zone volume. To calculate the ΔALV and Δsegment II/III, we subtracted the volume at < 24 h after ablation of the volume > 9 weeks after the ablation.

### Statistical Analysis

Baseline patient characteristics were described using descriptive statistics. The differences between baseline patient characteristic were tested: continuous variables were compared using the independent-sample *t* test or Mann–Whitney *U* test, depending on the normality. Histograms were used for checking normality. The Chi-squared or Fisher exact were used for categorical and dichotomous parameters. The liver volumes are checked for normality using histograms and compared with a Wilcoxon signed rank test. Data of the liver volumes were presented as median with interquartile range [IQR], or as percentage with IQR. The Spearman’s rho was calculated to compare the correlation between ablation volume and liver volumes. A *p* value of < 0.05 was considered statistically significant. All analyses were performed using IBM SPSS Statistics software version 25.0.

## Results

### Patient and Tumour Characteristics

**Fig. 1:**
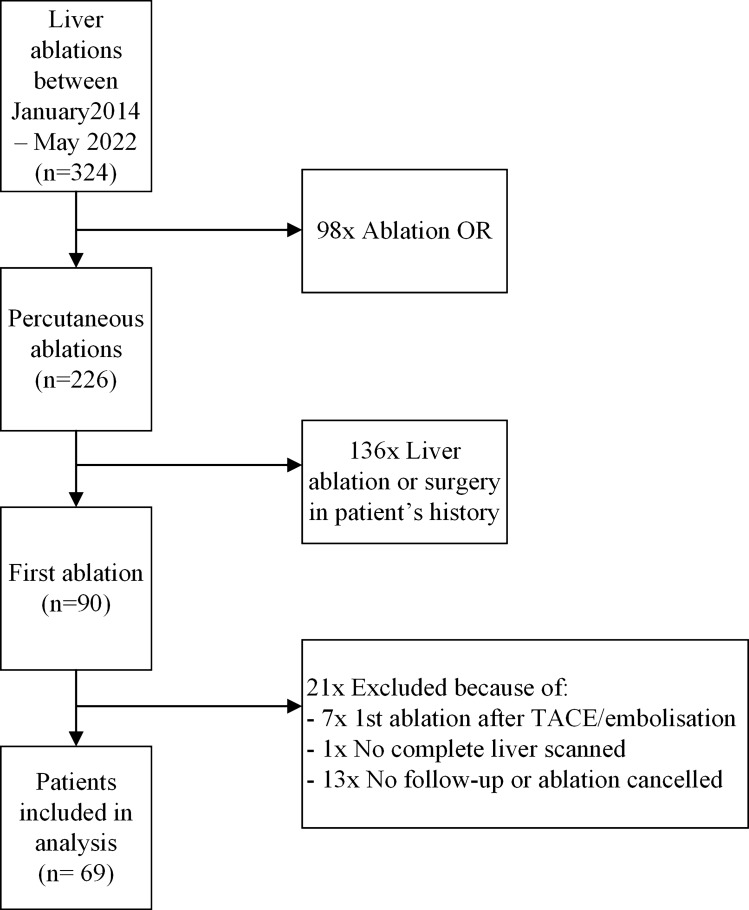
Flowchart of included patients with primary or secondary liver tumours. OR: operation room; TACE: transarterial chemoembolization.

Sixty-nine patients were included in the study (Fig. 1), 48 (69.6%) were male. Forty-three patients (62.3%) were treated for primary liver tumours and 26 patients for secondary liver tumours. Thirty-seven patients with primary liver tumours and none of the patients with secondary liver tumours had underlying liver cirrhosis. In total 89 tumours were treated, of which 53 lesions were primary liver tumours (59.6%) and 36 lesions were secondary liver tumours. In 11 patients two liver tumours were treated, in three patients three liver tumours, and in one patient four liver tumours. Patient characteristics are shown in Table 1. The TLV, ALV and the ablation zone volumes less than 24 h after ablation and after more than 9 weeks are presented in Table 2.

**Table 1:** Patients baseline characteristics

	Primary tumours (*n* = 43) *n*	Secondary tumours (*n* = 26) *n*
Age in years; mean (SD)	67.35 (8.41)	69.42 (10.42)
BMI in kg/m^2^; mean (SD)	29.12 (5.41)	26.52 (3.65)
Sex, male	33	15
*Portal hypertension*		
Yes	37	3
No	6	23
*Cirrhosis*		
Yes	37	0
No	6	26
*Child–Pugh score of cirrhotic patients*^*#*^		
A	35	NA
B	6	NA
C	1	NA
*Neo-adjuvant chemotherapy*		
Yes	4	12
No	33	11
Unknown	6	3
*ASA score*		
2	13	20
3	25	6
4	5	0
Total tumours^*^		
Subcapsular	27	14
Located deep	26	22
Total liver volume in cm^3^;	1591.92	1492.01
median [IQR]	[1418.20–1943.41]	[1232.34–1921.59]
Segment II + III volume in cm^3^; median [IQR]	368.18 [308.25–459.01]	248.58 [188.09–311.07]

BMI: body mass index; ASA score: American Society of Anesthesiologists score

^#^In one patient, the Child–Pugh score was unavailable. *Eleven patients were treated for two liver tumours, three patients for three liver tumours and one patient for four liver tumours

**Table 2:** Liver volume after liver ablation

	Primary tumours			Secondary tumours		
	Imaging < 24 h after ablation Median (IQR) (*N* = 43)	Imaging > 9 weeks after ablation Median (IQR) (*N* = 43)	*p* value	Imaging < 24 h after ablation Median (IQR) (*n* = 26)	Imaging > 9 weeks after ablation Median (IQR) (*n* = 26)	*p* value
TLV in cm^3^	1626.14 (1393.12–1902.50)	1591.92 (1418.20–1943.41)	0.433	1449.69 (1180.46–1790.74)	1492.01 (1232.34–1921.59)	0.058
%TLV	100.00	99.18 (93.26–107.91)	0.579	100.00	104.55 (97.77–110.54)	0.101
ALV in cm^3^	1597.57 (1380.49–1866.71)	1576.82 (1411.46–1935.40)	0.744	1392.32 (1158.22–1764.21)	1477.47 (1227.85–1911.73)	0.010
%ALV	100.00	98.72 (92.99–108.35)	0.856	100.00	106.87 (99.66–113.03)	0.016
Segment II/III in cm^3^	373.12 (310.48–494.38)	368.18 (308.25–459.01)	0.655	241.44 (170.82–270.74)	248.58 (188.09–311.07)	0.002
%Segment II/III	100.00	100.43 (92.85–109.41)	0.699	100.00	105.81 (100.06–115.65)	0.003
Ablation volume in cm^3^	34.56 (22.25–50.24)	22.08 (12.87–40.04)		37.99 (18.07–60.05)	13.48 (9.00–31.16)	
%Ablation volume	100.00	67.54 (47.50–78.19)		100.00	54.58 (35.83–69.07)	

TLV: Total liver volume; ALV: Absolute liver volume

### Liver Volumes—Total Liver Volume

The median TLV in patients with primary liver tumours showed a slight decrease from 1626.14 cm^3^ to 1591.92 cm^3^ (*p* = 0.433), the median TLV in patients with secondary liver tumours showed a slight increase from 1449.69 cm^3^ to 1492.01 cm^3^ (*p* = 0.058), although both not statistically significant. The median percentage in patients with secondary liver tumours increased to 104.55% [IQR = 97.77–110.54%].

### Liver Volumes—Ablation Volume

The ablation volume decreased in both groups. In patients with primary liver tumours, the median ablation volume decreased from 34.56cm^3^ to 22.08 cm^3^ (67.54% [IQR = 78.19–47.50%]). In patients with secondary liver tumours, the median volume of the ablation zone decreased from 37.99 cm^3^ to 13.48 cm^3^, reflected in a decrease of the median percentage to 54.58% [IQR = 69.07–35.83%].

### Liver Volumes—Absolute Liver Volume

Median ALV remained stable in patients with primary liver tumours (1597.57 cm^3^ before versus 1576.82 cm^3^ after (*p* = 0.856)). Median ALV in cm^3^ and percentage statistically significantly increased in patients with secondary liver tumours from 1392.32.45 cm^3^ to 1477.47 cm^3^ (*p* = 0.010) and to 106.87% ([IQR = 99.66–113.03%], *p* = 0.016), respectively, compared to baseline liver volumes. No statistically significant correlation was measured between ALV and the ablation volume in both groups (Table 3).

**Table 3:** Correlation outcomes in patients with primary and secondary liver tumours

	Primary tumours	Secondary tumours		
	Spearman’s rho correlation	*p* value	Spearman’s rho correlation	*p* value
Ablation volume vs. ΔALV*	− 0.178	0.253	0.182	0.373
Ablation volume vs. ΔSegment II/III	− 0.186	0.232	0.393	0.047

* ΔALV: Absolute liver volume > 9 weeks minus absolute liver volume < 24 h after ablation; † ΔSegment II/III: Segment II/III liver volume > 9 weeks minus segment II/III liver volume < 24 h after ablation

### Liver Volumes—Segments II/III

In patients with primary liver tumours, median volume of the distant liver segments/segments II/III remained stable with a slight, non-significant volume decrease from 373.812cm^3^ to 368.18cm^3^ (*p* = 0.655). A statistically significant increase in volume of segment II/III was seen in patients with secondary liver tumours, with an increase of the median from 241.44cm^3^ to 248.58cm^3^ and a percentage of 105.81% ([IQR = 100.06–115.65%], *p* = 0.002). A statistically significant correlation of *ρ* = 0.393 was found between Δsegment II/III and the ablation volume in patients with secondary liver tumours (*p* = 0.047).

## Discussion

This study showed a slight, but statistically significant increase in ALV and increase in volume of liver segments II/III after percutaneous ablation in patients with secondary liver tumours. In patients with primary liver tumours, the absolute liver volume and segment II/III remain unchanged.

To our knowledge, there are limited studies reporting on liver volume changes after percutaneous ablation. As mentioned above, early research in mice has demonstrated that thermal (radiofrequency) ablation leads to regeneration of liver volume [6]. Additionally, another mice study showed that hepatocytes around the ablation zone were positive for proliferation marker (CDC47) 72 h after ablation, whereas the hepatocytes at a larger distance (untreated lobe) from the ablation zone showed lower CDC47 levels [13], which was correlated with less hepatocyte proliferation. After 7 and 14 days, CDC47 levels were higher in the untreated lobe, suggesting a diffuse growth effect of hepatocytes after ablation in livers. In the current study, a similar growth in total liver volume but also in the volume of segments II/III (distant liver segments and surrogate for FLR) was observed in patients with secondary liver tumours, confirming a more diffuse growth effect. This is further underlined by the observation of a significant correlation between the growth of segment II/III and the size of the ablation volume.

Currently, different research groups focus on minimal invasive interventions to induce liver hypertrophy prior to major liver resection, in order to minimise the chance of PHLF [5, 10–12]. The current standard procedure to induce hypertrophy of the future liver remnant (FLR) is the portal venous embolization (PVE). It is, however, known that still 20–30% of the patients who have undergone a PVE in the preoperative phase never undergo liver resection [5, 12]. Insufficient FLR growth or progressive disease in the time between PVE and resection are two of the main reasons [14]. Combined portal and hepatic vein embolization (PVE/HVE) has shown higher rates of liver hypertrophy and resection compared to PVE alone, without an added risk of complications and mortality [15–17]. However, even with this promising and powerful technique, liver hypertrophy is not always sufficient or fast enough. Adding thermal ablation to this procedure might even further increase and speed up the hypertrophy rate. As the gain of volume, observed in our study, is relatively small (a volume increase of 6% of the absolute liver volume represents about 85cm^3^) thermal liver ablation on its own, with the purpose as a liver hypertrophy inducing procedure, is not feasible. Because of the observed systemic rather than a local effect, our findings, however, do suggest that thermal ablation done even outside the segments of the FLR could be of added value to other hypertrophy inducing techniques.

Data on thermal ablation in combination with an FLR hypertrophy inducing procedure are scarce. PVE in combination with RFA and “percutaneous microwave ablation liver partition and portal vein embolization (PALPP)”, which is a less invasive ALPPS procedure, has shown promising results. However, exact influence on outcomes needs to be further evaluated [18, 19]. It is important to mention that the use of RFA in these studies was not to induce liver hypertrophy but was seen as an additional observation after treating a liver tumour.

In contrast to patients with secondary liver tumours, hypertrophy was not seen in patients with primary liver tumours. These patients often presented with an underlying cirrhosis and the regeneration capacity of cirrhotic livers are often diminished [20]. Since none of the patients with secondary liver tumours in this cohort had underlying cirrhosis, these patients showed a higher regeneration capacity, which resulted in the significant increase in ALV.

The observation of liver growth after ablation also might indicate that larger ablation volumes and wider ablation margins should be possible, especially in patients with secondary liver tumours. Most treating physicians try to achieve a minimal ablation margin of 10 mm to the tumour in patients with secondary liver tumours, but may feel hampered in achieving a proper margin, by the possibility of inducing liver failure when treating larger or multiple (e.g. > 3) tumours in multiple sessions. Our results might stimulate physicians practising liver tumour ablation to adapt to a more aggressive ablation strategy (in patients with secondary liver tumours), aimed at lower local recurrences. The exact relation between ablation margin and oncological outcomes is, however, still under investigation [21–23].

Certain limitations of this study need to be acknowledged. First, this is a retrospective analysis with a limited number of included patients, which may have influenced the obtained results. However, due to the strict selection of consecutively included treatment naïve patients with imaging available on two specified timepoints, a homogenous group was selected to minimise selection bias. Future studies with larger patient groups and collecting other data (such as biomarkers) should therefore be performed to further assess the results.

Secondly, the liver analysis and volumetry were performed using two different volumetry programmes, as it was impossible to perform volumetry on MRI scans in the programme initially chosen (Syngo.via). Therefore, the Osirix DICOM viewer was added for the volumetry on MRI scans. However, to investigate the different performances of these software, five livers were measured by the same researcher with both software, no significant differences were observed. Thirdly, the patients in this study underwent ablations as local treatment for their tumours and not with the aim to induce hypertrophy. Therefore, liver hypertrophy should be considered as additional effect of the procedure and not as the primary intention.

## Conclusion

In patients with secondary liver tumours, ALV and segments II/III increased after MWA/RFA by an average of approximately 6%, while ALV in patients with primary liver lesions remained unchanged. Besides, the curative intent, these findings indicate the potential added benefit of thermal liver ablation on FLR hypertrophy inducing procedures in patients with secondary liver lesions.

## References

[1] Rhim H, Lim HK (2010). Radiofrequency ablation of hepatocellular carcinoma: pros and cons. Gut Liver.

[2] Meloni MF, Chiang J, Laeseke PF, Dietrich CF, Sannino A, Solbiati M (2017). Microwave ablation in primary and secondary liver tumours: technical and clinical approaches. Int J Hyperthermia.

[3] Izzo F, Granata V, Grassi R, Fusco R, Palaia R, Delrio P (2019). Radiofrequency ablation and microwave ablation in liver tumors: an update. Oncologist.

[4] Jin S, Fu Q, Wuyun G, Wuyun T (2013). Management of post-hepatectomy complications. World J Gastroenterol.

[5] Ni JY, Xu LF, Sun HL, Zhou JX, Chen YT, Luo JH (2013). Percutaneous ablation therapy versus surgical resection in the treatment for early-stage hepatocellular carcinoma: a meta-analysis of 21,494 patients. J Cancer Res Clin Oncol.

[6] Ypsilantis P, Pitiakoudis M, Souftas VD, Lambropoulou M, Tsalikidis C, Foutzitzi S (2008). Liver regeneration following radiofrequency ablation. J Surg Res.

[7] Abulkhir A, Limongelli P, Healey AJ, Damrah O, Tait P, Jackson J (2008). Preoperative portal vein embolization for major liver resection: a meta-analysis. Ann Surg.

[8] Schreckenbach T, Liese J, Bechstein WO, Moench C (2012). Posthepatectomy liver failure. Dig Surg.

[9] van Lienden KP, van den Esschert JW, de Graaf W, Bipat S, Lameris JS, van Gulik TM (2013). Portal vein embolization before liver resection: a systematic review. Cardiovasc Intervent Radiol.

[10] Yokoyama Y, Nagino M, Nimura Y (2007). Mechanisms of hepatic regeneration following portal vein embolization and partial hepatectomy: a review. World J Surg.

[11] Schadde E, Ardiles V, Robles-Campos R, Malago M, Machado M, Hernandez-Alejandro R (2014). Early survival and safety of ALPPS: first report of the International ALPPS Registry. Ann Surg.

[12] Sandstrom P, Rosok BI, Sparrelid E, Larsen PN, Larsson AL, Lindell G (2018). ALPPS improves resectability compared with conventional two-stage hepatectomy in patients with advanced colorectal liver metastasis: results from a scandinavian multicenter randomized controlled trial (LIGRO Trial). Ann Surg.

[13] Rozenblum N, Zeira E, Bulvik B, Gourevitch S, Yotvat H, Galun E (2015). Radiofrequency ablation: inflammatory changes in the periablative zone can induce global organ effects, including liver regeneration. Radiology.

[14] Cassese G, Han HS, Lee B, Cho JY, Lee HW, Guiu B (2022). Portal vein embolization failure: current strategies and future perspectives to improve liver hypertrophy before major oncological liver resection. World J Gastrointest Oncol.

[15] Heil J, Korenblik R, Heid F, Bechstein WO, Bemelmans M, Binkert C (2021). Preoperative portal vein or portal and hepatic vein embolization: DRAGON collaborative group analysis. Br J Surg.

[16] Korenblik R, Olij B, Aldrighetti LA (2022). Dragon 1 protocol manuscript: training, accreditation, implementation and safety evaluation of portal and hepatic vein embolization (PVE/HVE) to accelerate future liver remnant (FLR) hypertrophy. Cardiovasc Intervent Radiol.

[17] Korenblik R, van Zon JFJA, Olij B (2022). Resectability of bilobar liver tumours after simultaneous portal and hepatic vein embolization versus portal vein embolization alone: meta-analysis. BJS open.

[18] Wang Q, Ji Y, Brismar TB, Chen S, Li C, Jiang J (2021). Sequential portal vein embolization and percutaneous radiofrequency ablation for future liver remnant growth: a minimally invasive alternative to ALPPS Stage-1 in treatment of hepatocellular carcinoma. Front Surg.

[19] de Hong F, Zhang YB, Peng SY, Huang DS (2016). Percutaneous microwave ablation liver partition and portal vein embolization for rapid liver regeneration: a minimally invasive first step of ALPPS for hepatocellular carcinoma. Ann Surg.

[20] Aierken Y, Kong LX, Li B, Liu XJ, Lu S, Yang JY (2020). Liver fibrosis is a major risk factor for liver regeneration: a comparison between healthy and fibrotic liver. Medicine (Baltimore).

[21] Oosterveer TTM, van Erp GCM, Hendriks P, Broersen A, Overduin CG, van Rijswijk CSP (2022). Study protocol PROMETHEUS: prospective multicenter study to evaluate the correlation between safety margin and local recurrence after thermal ablation using image co-registration in patients with hepatocellular carcinoma. Cardiovasc Intervent Radiol.

[22] Lencioni R, de Baere T, Martin RC, Nutting CW, Narayanan G (2015). Image-guided ablation of malignant liver tumors: recommendations for clinical validation of novel thermal and non-thermal technologies–A western perspective. Liver Cancer.

[23] Wang X, Sofocleous CT, Erinjeri JP, Petre EN, Gonen M, Do KG (2013). Margin size is an independent predictor of local tumor progression after ablation of colon cancer liver metastases. Cardiovasc Intervent Radiol.

